# Anti-PD-1 combined with hypomethylating agent and CAG regimen bridging to allogeneic hematopoietic stem cell transplantation: a novel strategy for relapsed/refractory acute myeloid leukemia

**DOI:** 10.3389/fimmu.2024.1409302

**Published:** 2024-08-16

**Authors:** Yu-Xin Wang, An Wang, Yong-Feng Su, Jun Wang, Yu-Hang Li, Fei Li, Yu Jing, Lei Xu, Yi-Zhi Wang, Xuan Zheng, Chun-Ji Gao, Liang-Ding Hu, Xiao-Ning Gao, Dai-Hong Liu

**Affiliations:** ^1^ Senior Department of Hematology, the Fifth Medical Center of PLA General Hospital, Beijing, China; ^2^ Graduate School, Chinese PLA General Hospital, Beijing, China

**Keywords:** Anti-PD-1, allogeneic hematopoietic stem cell transplantation, relapse/refractory acute myeloid leukemia, acute graft-versus-host disease, posttransplant cyclophosphamide

## Abstract

**Introduction:**

The prognosis of relapsed/refractory acute myeloid leukemia (r/rAML) is dismal, and allogeneic hematopoietic stem cell transplant (allo-HSCT) is a potential cure. Combining anti-PD-1, hypomethylating agent (HMA), and CAG (cytarabine, aclarubicin/idarubicin, granulocyte colony-stimulating factor) regimen has showed primary efficacy in r/rAML. However, pre-transplant exposure to anti-PD-1 may lead to severe graft-versus-host disease (GVHD). This preliminary study aimed to evaluate the safety and efficacy of allo-HSCT in r/rAML patients receiving the anti-PD-1+HMA+CAG regimen.

**Methods:**

Fifteen r/rAML patients (12 related haploidentical donors [HIDs], 2 matched siblings, 1 unrelated donor) received this regimen and subsequent peripheral blood HSCT.

**Results:**

Four patients with HIDs received a GVHD prophylaxis regimen consisted of Anti-thymocyte globulin and a reduced-dose of post-transplant cyclophosphamide. The median follow-up was 20.9 months (range, 1.2-34.2). The cumulative incidences of acute GVHD grade 2-4 and grade 3-4 were 40% and 13.3%, respectively. The 2-year incidence of moderate-to-severe chronic GVHD, non-relapse mortality, and relapse were 10%, 22.3%, and 22.5%, respectively. The 2-year overall survival and GVHD-free/relapse-free survival rates were 54% and 48.6%, respectively. No death or relapse was observed in the PTCy group.

**Conclusion:**

The anti-PD-1+HMA+CAG regimen bridging to allo-HSCT for r/r AML was tolerable with promising efficacy. GVHD prophylaxis with PTCy for HID-HSCT showed preliminary survival advantage.

## Introduction

The prognosis of relapsed/refractory acute myeloid leukemia (r/rAML) is dismal with conventional multi-agent chemotherapy and allogeneic hematopoietic stem cell transplant (allo-HSCT) is a potential cure (1). Nevertheless, relapsed/refractory status prior to the start of transplantation remains an independent risk for relapse after transplantation. Programmed cell death protein1 (PD-1) and its PD-1 ligand 1 (PD-L1) are essential immune checkpoint mechanisms that maintain immune tolerance (2). However, when hijacked by tumor cells, the expression of PD-L1 on tumor cells and tumor-associated antigen-presenting cells changes, suppressing PD-1-expressing T cells to evade immunosurveillance and cause immune evasion (3, 4). PD-1 blockade induces preferential stimulation of antitumor effector T cells and mediates antitumor activity (2). Consequently, anti-PD-1 therapies have shown substantial clinical efficacy in the treating hematologic malignancies, such as classic Hodgkin lymphoma (HL), non-HL, and multiple myeloma (5). Similarly, PD-1 dysregulation has been observed in AML (6, 7), leding to clinical trials evaluating PD-1 blockade therapy in AML patients. Furthermore, patients with multiple relapses have higher frequencies of PD-1^+^/CD8^+^ T cells in bone marrow (BM) compared to those with first relapse or newly diagnosed AML (8), and this overexpression of PD-1 may predict poorer overall survival (OS) (9, 10). Although anti-PD-1 monotherapy exhibited limited clinical efficacy, combinations of anti-PD-1 and hypomethylating agent (HMA) or cytotoxic chemotherapy have been investigated for r/r AML in recent years (11–13). In our recent study, combinations of azacitidine or decitabine plus CAG regimen (cytarabine, aclarubicin, granulocyte-colony-stimulating factor [G-CSF]) with tislelizumab have showed primary efficacy in r/rAML patients (14). Consequently, the participants in these anti-PD-1-based regimen trials are increasingly becoming potential candidates for allo-HSCT with curative intent.

However, the transplant outcomes of HSCT may be altered in patients previously exposed to PD-1 inhibitors due to their immunomodulatory mechanisms and long half-lives (15, 16). Specifically, residual PD-1 inhibitors at the time of HSCT may lead to a more robust donor T cell response, resulting in enhanced graft-versus-tumor (GVT) effects, but on the downside, this may also increase toxicity in the form of graft-versus-host disease (GVHD) (15, 16). A mechanism study using murine models demonstrated that inhibition of PD-1 signaling induced aggressive expansion of CD4^+^ conventional T cells, while regulatory T cells could not maintain expansion due to high susceptibility to apoptosis. This discordant immune recovery resulted in the development of severe GVHD (17). Initial clinical studies have shown that pretransplant exposure to checkpoint inhibitor (CPI) may lead to higher rates of grade 3-4 acute GVHD (aGVHD) (17% ~ 29%) and GVHD-related mortality (9% ~ 35%) than expected (18–21). Nevertheless, most of the previously reported evidence was based on lymphoma patients, with only a few on AML patients (13, 19, 22). Additionally, the baseline characteristics of patients in these reports were highly heterogeneous in terms of the allograft donor source, disease risk stage, GVHD prophylaxis type, and follow-up period, affecting the credibility of their transplant outcomes.

Recent studies have demonstrated that post-transplant cyclophosphamide (PTCy) may effectively mitigate GVHD risk associated with pretransplant CPI use (19, 23–25). In our conventional routine for GVHD prophylaxisis based on Ciclosporin A (CsA) plus short-term methotrexate (MTX), the incidences of acute GVHD grade 2-4, grade 3-4 and chronic GVHD were 50%, 9% and 25%, respectively. Our previous study showed promising efficacy of the novel regimen (anti-PD-1+HMA+CAG) as chemotherapy in 27 r/r AML patients (14). Therefore, this study aimed to evaluate the toxicity and efficacy of HSCT in r/r AML who received this novel regimen pretransplant. PTCy was added to the conventional GVHD prophylaxis in 4 out of 12 patients with haploidentical donors (HIDs).

## Methods

### Study design and participants

This retrospective study enrolled patients with r/r AML who had failed at least one cycle of intensive induction chemotherapy or developed relapse. The patients were treated with the tislelizumab + HMA + CAG regimen before undergoing HSCT at the Chinese PLA General Hospital in Beijing. From September 15, 2020, to November 30, 2023, a total of 41 patients received the tislelizumab + HMA + CAG regimen (ClinicalTrials.gov identifier NCT04541277) for the treatment of AML, and among them, 15 patients underwent allo-HSCT after receiving the treatment regimen (**Supplementary Figure 1**).

The trial was approved by the Ethics Committee of the Chinese PLA General Hospital (No. S2020-296-01) and conducted in accordance with the Declaration of Helsinki and the International Conference on Harmonization Good Clinical Practice guidelines. All participants provided written informed consent before enrollment.

### Definitions and assessments

The terms “treatment response” and “disease status” were redefined based on the 2022 ELN recommendations provided by an International Expert Panel for AML (26). Complete remission (CR) was defined as having less than 5% bone marrow blasts without any recurrence of extramedullary disease, along with complete hematologic recovery, in patients with AML. The criteria for CR with incomplete hematologic recovery (CRi), partial remission (PR), and morphologic leukemia-free state (MLFS) have not changed. No response (NR): patients evaluable for response but not meeting the criteria for CR, CR with partial hematologic recovery, CRi, MLFS or PR will be categorized as having ‘NR’ (26, 27). Refractory disease was defined as the absence of CR/CRi after one cycle of induction chemotherapy. On the other hand, relapse is diagnosed in AML patients who have previously achieved CR but demonstrate an increase in blasts in the bone marrow to 5% or more, reappearance of blasts in the blood, or the development of extramedullary disease. Both the disease typing diagnosis and the risk categorization were reassessed using the 2022 ELN recommendations (26, 28). HSCT-specific comorbidity index (HCT-CI) and Disease Risk Index (DRI) were calculated for all patients as described, respectively (29, 30). Detailed data were recorded in standardised electronic forms prospectively but revaluated and integrated retrospectively.

The endpoints of interest included cumulative incidence of aGVHD (100 days) and chronic GVHD (cGVHD) (2-year), 2-year cumulative incidence of non-relapse mortality (NRM), 2-year cumulative incidence of relapse (CIR), the probability of 2-year overall survival (OS), and 2-year GVHD-free/relapse-free survival (GRFS). The criteria defined by Przepiorka et al. are used for the diagnosis and grading of aGVHD, while the NIH scoring system is used for cGVHD diagnosis and grading (31). NRM was defined as death from any cause other than malignancy relapse. CIR was measured from the date of transplantation until the date of hematologic relapse. The time from transplantation to any cause death or the last follow-up was defined as OS. GRFS events were defined as grade 3–4 aGVHD or cGVHD requiring systemic immunosuppressive treatment, disease relapse, or any-cause death during the first 12 months after allogeneic HCT (32). The first three consecutive days with an absolute neutrophil count > 0.5 × 10^9^/L indicated neutrophil recovery. Platelet recovery was defined as the first seven days with an untransfused platelet count of > 20 × 10^9/^L.

### Tislelizumab + HMA + CAG regimen

The tislelizumab + HMA + CAG regimen was initiated as described previously (14). The anti-PD-1 containing treatment regimen pretransplant consisted of azacitidine 75 mg/m^2^ subcutaneously (SQ) daily, day 1-7 or decitabine 20 mg/m^2^ intravenously (i.v.) daily, day 1-5 plus CAG regimen (cytarabine 100 mg i.v. every 12 h, day 1–5; aclarubicin 20 mg i.v. daily, day 1-5 or idarubicin 10 mg i.v., day 1, 3 and 5); and G-CSF 5 μg/kg/day subcutaneously (SC), from day 0 to end of chemotherapy (when the white blood cell count exceeds 10× 10^9^/L) with tislelizumab 200 mg i.v., day 6 or day 8 (started the day after chemotherapy was stopped). Dosage of cytarabine was reduced to 10 mg SC every 12 h in patients with any of the following conditions: (1) presence of obvious heart, lung and kidney complications; (2) BM hypoproliferation. Each cycle lasted for 28 days and the cycles were repeated every 4–6 weeks, depending on count recovery and in the absence of disease progression or unacceptable toxicity.

### Conditioning regimens and GVHD prophylaxis

All recipients received myeloablative conditioning, which included the following regimens:

1. Modified busulfan plus Cy (BuCy) based regimen: It consisted of Bu (9.6 mg/kg, i.v., days -10 to -8), carmustine, (250 mg/m^2^, day -5), cytarabine (4 g/m^2^, days -7 to -6), and Cy (100 mg/kg, days -4 to -3).
2. Modified fludarabine (Fl) and Bu-based regimen: In this regimen, Cy in the BuCy regimen was replaced with Fl (30 mg/m^2^, days -7 to -3).

For basic GVHD prophylaxis, CsA, mycophenolate mofetil (MMF), and short-term MTX were considered. CsA (3 mg/kg, i.v., q12 h) was administered starting on day -10, and the trough concentration was adjusted to 150~250 ng/ml. CsA dose was decreased from 3 months after HSCT in case of no GVHD occurrence until discontinuation at around 1.5 years. MMF was administered orally starting on day -10 (0.5 g, q12 h), and withdrawn on day +45 for HID-HSCT and +30 for matched sibling donors (MSD)-HSCT. MTX was administered i.v. with a dose of 15 mg/m^2^ on day +1 and 10 mg/m^2^ on days +3, +6, and +11. Anti-thymocyte globulin (ATG, 5 mg/kg, days -3 to -2) was used in MSD-HSCT while ATG (1.5 mg/kg, day -5; 2.5 mg/kg, day -4) was used in both HID-HSCT and unrelated donor (URD)-HSCT. The mathematical function was exploited to determine the total targeted ATG dose on days -3 to -2 based on the concentrations of active ATG on days -5 to -4 (33). Subgroup analysis in HID-HSCT was stratified by GVHD prophylaxis based on the use of reduced-dose PTCy (two doses of 14.5 mg/kg Cy given on days +3 and +4 post-HSCT) (n=4) or non-PTCy (n=8). The supportive care strategies followed previously reported data (34).

Eligible responders were also offered allo-HSCT based on the availability of a suitable donor and at the discretion of the treating physician.

### Statistical analysis

The Kaplan–Meier method was used to calculate OS and GRFS. Cumulative rates of NRM, relapse, and GVHD were estimated using the competing risk model. Competing events were defined as follows: for NRM, relapse; for relapse, NRM; for GVHD, death without GVHD; incidence of various transplantation-related complications, death without various transplantation-related complications. Statistical analyses were performed using NCSS 2021 (64-bit), RStudio (Version, 6.2.0), and GraphPad Prism (Version, 9.2.0.) software tools.

## Results

### Characteristics of patients, pre-transplant treatment, and transplantation

**Figure 1:**
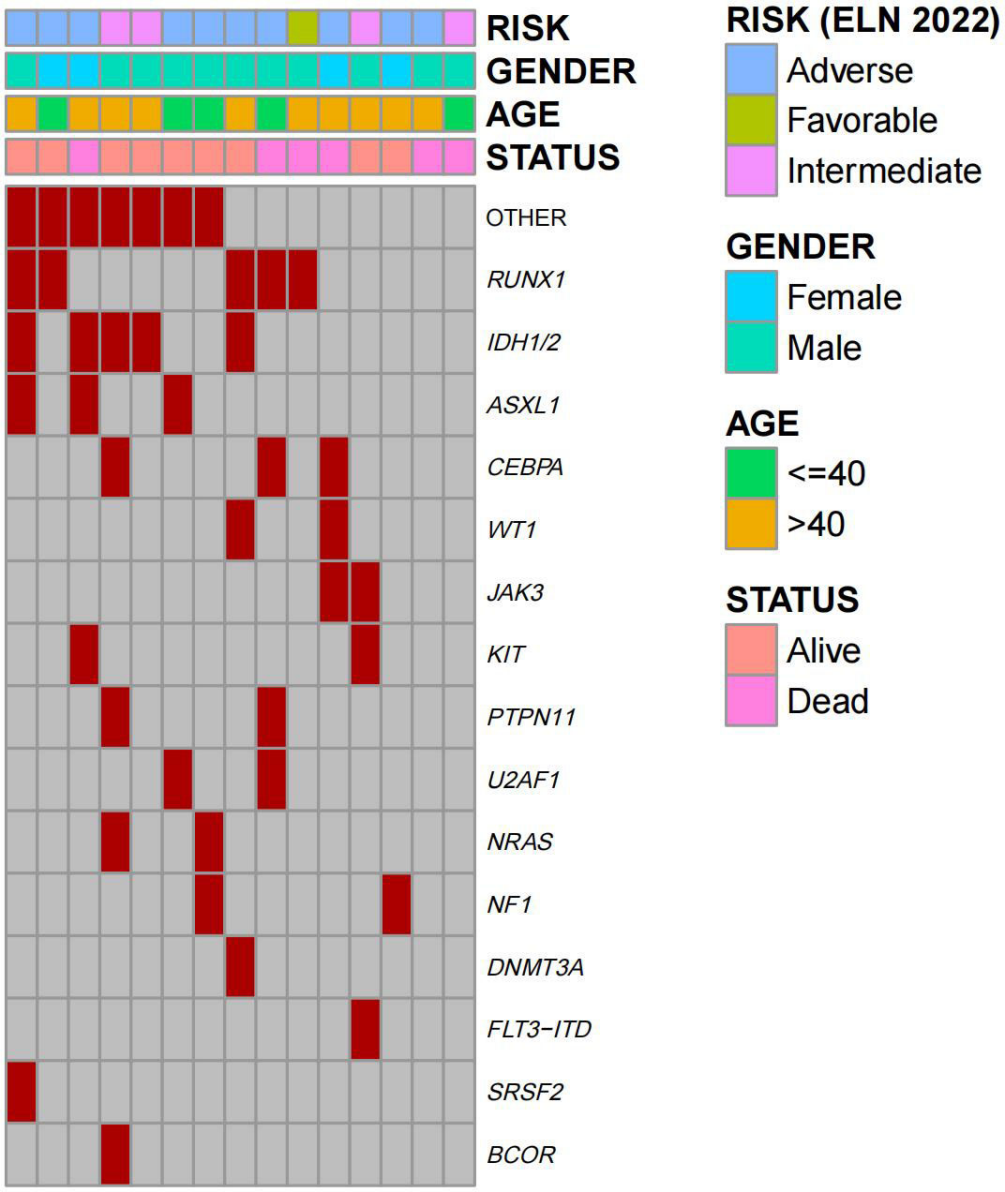
Heatmap of basic clinical traits and somatic mutations of patients (n=15). Columns represent individual patients and rows represent clinical variables or the presence of mutations identified at diagnosis.

A total of 15 (male:11; female: 4) r/r AML patients treated with tislelizumab + HMA + CAG regimen prior to HSCT were enrolled in this analysis. Baseline demographic and clinical characteristics are shown in **Table 1**. The median age of the patients at diagnosis was 42 years (range, 21-56 years). Among 15 patients, 11 (73.3%) patients had refractory disease while 4 (26.7%) patients had disease relapse. A heatmap of basic clinical traits and somatic mutations of 15 patients is presented in **Figure 1**. The median number of the 3 systematic treatments pre-transplant was 3 (range 2-7) and 1 cycle (range, 1-4) for the tislelizumab + HMA + CAG regimen. As for immune-related adverse events (irAEs), only two patients were of grade 3 (thyroiditis and pneumonitis). No deaths were directly attributable to irAEs. The best response to tislelizumab + HMA + CAG regimen was CR/CRi in 11 patients (73.3%; CR:10; CRi:1), PR in 1 patient (6.7%), and NR in 3 patients (20%). Prior to HSCT, 73.3% of the patients were in CR (measurable residual disease (MRD)+:7; MRD-:4) and 26.7% had refractory disease (PR:1; NR:3). Of them, ten patients (66.7%) achieved CR/CRi after 1-2 courses of tislelizumab + HMA + CAG regimen, and another patient (6.7%) achieved CR after 4 courses (**Table 1**).

**Table 1:** Baseline and transplant characteristics of patients (n=15).

Characteristic	Number	Percent
Total (cases)	15	100.0
Patient age (median, years [range])	42 [21–56]	–
Sex		
Male	11	73.3
White blood cell count (median, ×10^9^/L [range])	11 [1.6-156]	–
ELN 2022 disease typing diagnosis		
AML with recurrent genetic abnormality	7	46.7
AML with MR gene mutations	2	13.3
AML with MR cytogenetic abnormalities	3	20.0
AML not otherwise specified	3	20.0
Diagnostic qualifiers		
AML-*de novo*	13	86.7
Therapy-related AML	2	13.3
With extramedullary leukemia	**2**	**13.3**
ELN 2022 risk classification		
Favorable	1	6.7
Intermediate	4	26.7
Adverse	10	66.7
Fusion genes		
AML1-ETO	5	33.3
MLL-AF6	1	6.7
MLL-AF10	1	6.7
NUP98-NSD1	1	6.7
Cycles of prior systemic therapies (median [range])	3 [2–7]	–
Anti-PD-1 treatment regimen		
Tislelizumab + ACAG	7	46.7
Tislelizumab + DCAG	8	53.3
Number cycles of Tislelizumab + HMA + CAG treatment (median [range])	1 [1–4]	–
Best response to PD-1 inhibitor		
CR/CRi	11	73.3
PR	1	6.7
NR	3	20.0
Number of patients with irAE while on PD-1 inhibitor (prior to transplant)		
Immune-related thyroiditis	1	6.7
Immune-related pneumonitis	1	6.7
Interval between last anti-PD-1 treatment and HSCT (median, months [range])	2.5 [1.7-6.9]	–
Donor/HLA type		
Matched related	2	13.3
Haploidentical	12	80.0
Mismatched unrelated	1	6.7
Donor age (median, years [range])	31 [13–59]	–
Donor/recipient sex		
Female to male	4	26.7
Others	11	73.3
ABO compatibility		
Matched	8	53.3
Major mismatched	2	13.3
Minor mismatched	2	13.3
Major and minor mismatched	3	20.0
HCT-CI scores before HSCT		
<3	13	86.7
≥3	2	13.3
DRI before HSCT		
Low	2	13.3
Intermediate	9	60
High	3	20
Very high	1	6.7
ECOG		
0	1	6.7
1	11	73.3
2	3	20.0
Conditioning regimen		
Modified Bu/Cy based regimen	13	86.7
Modified Fl/Bu based regimen	2	13.3
GVHD prophylaxis		
ATG, CSA, MMF, MTX, PTCy	4	26.7
ATG, CSA, MMF, MTX	11	73.3
The volume stem cell infusion		
Mononuclear cells (median, ×10^8^/L [range])	13.5 [5.7-28.2]	–
CD34^+^ cells (median, ×10^6^/L [range])	3.6 [1.4-8.7]	–
Follow-up from transplantation (median, months [range])	20.9 [1.2-34.2]	–

ELN, European Leukemia Net; AML, Acute myeloid leukemia; MR, myelodysplasia-related; ACAG, azacytidine, cytarabine, aclarubicin, G-CSF; DCAG, decitabine, cytarabine, aclarubicin, G-CSF; CR, complete remission; CRi, CR with incomplete hematologic recovery; PR, partial remission; NR, no response; irAE: immune-related adverse events; HCT -CI, hematopoietic cell transplant-comorbidity index; HSCT, hematopoietic stem cell transplantation; DRI, Disease Risk Index; ECOG, ECOG, Eastern Cooperative Oncology Group performance status; Bu, busulfan; Cy, cyclophosphamide; Fl, fludarabine; TBI, total body irradiation; TT, thiotepa; ATG, anti-thymocyte globulin; CSA, cyclosporine; MTX, methotrexate; MMF, mycophenolate mofetil; PTCy, posttransplant cyclophosphamide.

The median interval between last anti-PD-1 treatment and allo-HSCT was 2.5 months (range, 1.7-6.9). **Table 1** also summaries the transplantation characteristics of 15 patients. Allo-HSCT following AML treatment was performed in 15 patients with HLA-HID (n = 12), HLA-MSD (n = 3), and HLA-URD (n = 1). All 15 patients received graft sources from hematopoietic stem cells of the donors’ peripheral blood. Patients were analyzed on the basis of whether they received PTCy as GVHD prophylaxis. The GVHD prophylaxis consisted of ATG + CSA + MTX + MMF in 11 patients (HID: 8; MSD: 2; URD:1) and ATG + CSA + MTX + MMF + PTCy in 4 patients (HID: 4).

### Engraftment and GVHD

**Figure 2:**
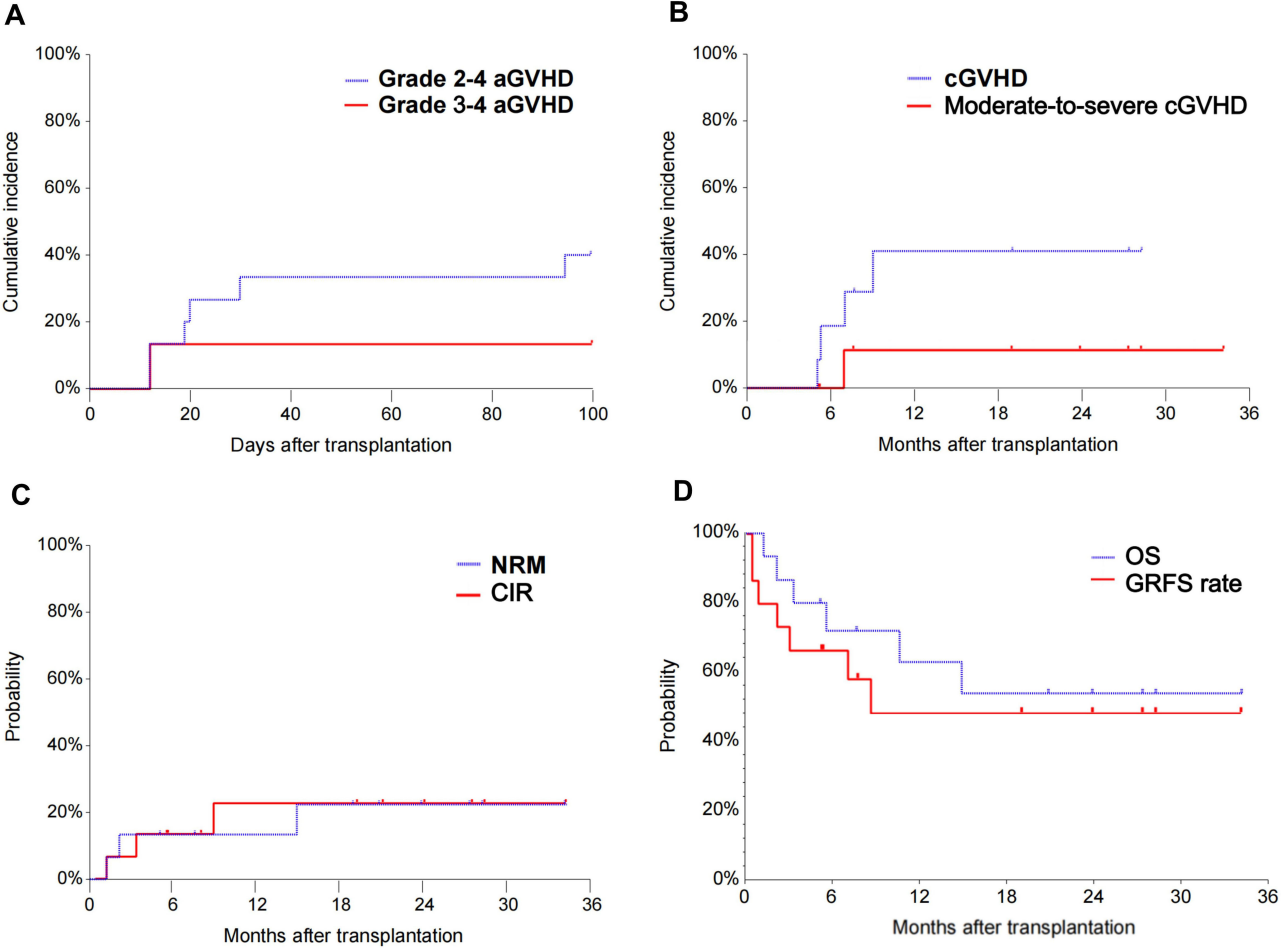
The cumulative incidence of grade 2-4 aGVHD and grade 3-4 aGVHD of patients enrolled **(A)**. The cumulative incidence of cGVHD and moderate-to-severe cGVHD of patients enrolled **(B)**. The NRM and CIR of patients enrolled **(C)**. The OS and GRFS of patients enrolled **(D)**. aGVHD, acute graft-versus-host disease; cGVHD, chronic graft-versus-host disease; NRM, non-relapse mortality; CIR, cumulative incidence of relapse; OS, overall survival; GRFS, graft-versus-host disease-free/relapse-free survival.

All patients (100%) had successful neutrophil engraftment while fourteen patients (93.3%) had successful platelet repopulation after a median of 14 days (range, 12–17 days) and 16 days (range, 11–25 days), respectively. Of the 15 transplanted patients, 7 (46.7%) developed graft-versus-host disease (n=5, grades 1–2, n=2, grade 3–4), and 6 responded to the treatment. The 100-day cumulative incidences of aGVHD, grade 2-4, and grade 3-4 aGVHD were 46.7%, 40%, and 13.3%, respectively (**Figure 2A**). Patient number 6 who failed to achieve platelet engraftment developed grade 3-4 aGVHD of the lower gastrointestinal tract on day 12, which was complicated by acute kidney injury, drug-induced liver injury, intracranial hemorrhage, gastrointestinal hemorrhage, and death on day 36. Patient number 8 developed grade 3 gastrointestinal aGVHD combined with grade 1 hepatic aGVHD and achieved CR after the treatment of Ruxolitinib (**Supplementary Table 1**). The 2-year incidence of cGVHD and moderate-to-severe cGVHD were 40.9% and 10%, respectively (**Figure 2B**).

### Toxicity and outcomes

The median follow-up time was 20.9 months (1.2-34.2 months) with a mortality rate of 40% (n=6). **Supplementary Table 1** demonstrates the toxicity, outcomes, and deaths of 15 patients during transplantation. Three patients died of nonrelapse-related conditions, one of grade 3-4 early aGVHD 4 with gut involvement after 1.2 months (patient 6), one of severe pulmonary infection after 2.1 months (patient number 12 was complicated by peritoneal effusion, transplant-associated thrombotic microangiopathy, multiple organ dysfunction syndrome), and another one had LOPS (late-onset severe pneumonia, LOSP) after 14.8 months (patient number 8) after transplantation. The cumulative incidence of cytomegalovirus (CMV) DNAemia in 2 years was 40% (95% CI: 22%-74%), and that of Epstein-Barr virus infection was 43% (95% CI: 16%-68%) (**Supplementary Table 1**). One patient developed CMV cystitis in +0.74 months and CMV pneumonia in +2.3 months.

**Figure 3:**
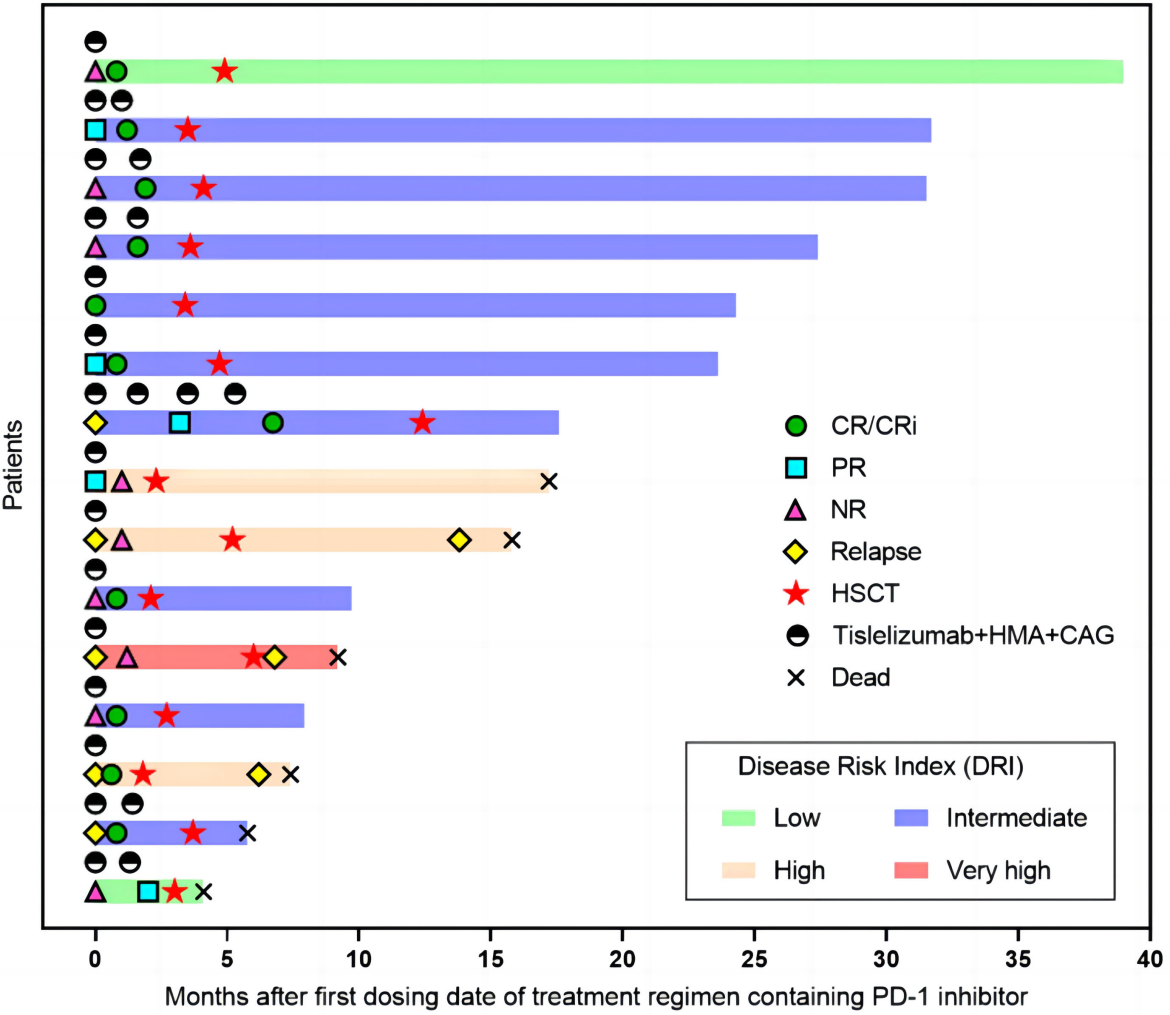
Swimmer plot illustrating the clinical course of 15 patients. Tislelizumab + HMA + CAG regimen, treatment response, survival status, and allogeneic stem cell transplantation status of the enrolled patients are shown. CR, complete remission; PR, partial remission; NR, no response; HSCT, hematopoietic stem cell transplantation; HMA, hypomethylating agent; CAG, azacytidine, cytarabine, aclarubicin.

One patient received Sorafenib as maintenance therapy 3 months after transplantation. Another patient received donor lymphocyte infusion three months after HSCT for relapse prophylaxis. Three patients died from relapse after 3.3, 5.5, and 10.6 months. The 2-year NRM and CIR were 22.3% (95% CI, 8.1-61.7) and 22.5% (95% CI, 8.1-62.2), respectively (**Figure 2C**). The 2-year OS and GRFS were 54% (95% CI, 26.1-81.9) and 48.6% (95% CI, 20.9-76.3) (**Figure 2D**), respectively. A total of 9 patients survived and remained in CR 5.2-39.2 months after transplantation (**Figure 3** **).**

### Subgroup analysis in HID-HSCT

In PTCy group, 3 patients developed aGVHD grade 1-2, and in non-PTCy group, 1 developed aGVHD grade 1-2 and 2 grade 3-4. One patient in each group develpoed cGVHD mild type. The median follow-up was 19 months in the non-PTCy group and 7.7 months in the PTCy group. In the non-PTCy group 3 patients died of NRM (n=2, pulmonary infection; n=1, grade 3-4 aGVHD) and 3 died of disease relapse; however no death or relapse was observed in the PTCy group. As observed in **Table 2**, the PTCy group showed a trend toward higher 2-year OS and GRFS rate as compared with the non-PTCy group.

**Table 2:** Transplantation outcomes of haploidentical transplantation by GVHD prophylaxis.

	All Patients N = 12		No PTCy Group N = 8		PTCy Group N = 4	
	Event/No.	Estimate (95% CI)	Event/No.	Estimate (95% CI)	Event/No.	Estimate (95% CI)
Acute GVHD (100-days)	6/12	50% (28.4%-88.0%)	3/8	37.5% (15.3%-71.7%)	3/4	75% (42.6%-100%)
Grade 1-2	4/12	33.3% (15.0-74.2%)	1/8	12.5% (2%-78.2%)	3/4	75% (42.6%-100%)
Grade 3-4	2/12	16.7% (4.7%-59.1%)	2/8	25% (7.5%-83%)	0/4	0%
Chronic GVHD (2-year)	2/9	29.6% (8.8%-99.9%)	1/5	20% (3.5%-100%)	1/4	50% (12.5%-100%)
Moderate-to-severe	0/9	0%	0/5	0%	0/4	0%
2-year OS	6/12	38.6% (6.1%-71.1%)	6/8	15.6% (0-43.4%)	0/4	100%
2-year CIR	3/12	30% (11.2%-80.5%)	3/8	41.7% (17.3%-100%)	0/4	0%
2-year NRM	3/12	29.5% (11.1%-79.2%)	3/8	40.6% (16.7%-98.7%)	0/4	0%
2-year GRFS rate	6/12	43.8% (11.3%-76.2%)	6/8	18.75% (0-49.7%)	0/4	100%

GVHD, graft-versus-host disease; OS, overall survival; CIR, cumulative incidence rate; NRM, non-relapse mortality; GRFS, GVHD-free/relapse-free survival.

## Discussion

The treatment of r/r AML patients is a known challenge for hematologists since decades. Despite numerous clinical studies, outcomes are consistently disappointing with 5-year OS rates reported to be not more than 10% (1). Traditional intensive chemotherapy for r/r AML mainly includes purine analogues (such as Fl and cladribine) -based regimens, with ORR of 30%-45% and median survival being 8-9 months (35). Recently, the combination of targeted therapy with chemotherapy has shown a higher remission rate, ranging from 40% to 70% in patients with relapsed/refractory AML, which has become a crucial option in clinical practice (36–40). A multicenter, phase 2 trial has shown that the regimen combining venetoclax (VEN) with azacitidine and homoharringtonine for 96 R/R AML patients achieved a high composite complete remission rate (CR plus CRi) of 70.8% (37). Notably, 40 patients (41.6%) who bridged to allo-HSCT achieved the 1-year OS of 85.0%. However, extended follow-up is essential to confirm the durability of the responses and long-term survival. Furthermore, a combination regimen of targeted drugs (including drugs for specific targets and VEN) ± HMA has also achieved good therapeutic effects for R/R AML patients (41–44).Our previous study reported that the combination of the anti-PD-1 antibody tislelizumab and HMA + CAG regimen improved ORR up to 63% (14), thereby providing patients with the suitable opportunity of allo-HSCT. Despite the robust efficacy of PD-1 inhibition, its immune mechanism raised high concerns of excessive T cell activation and immune toxicity like GVHD after allo-HSCT (16).

The primary observation from our investigation is that allo-HSCT after tislelizumab + HMA + CAG regimen appears feasible with an acceptable safety and efficacy profile, thereby providing a promising potential treatment strategy for r/r AML. Meanwhile, this study has utilized the combination of PTCy and ATG for GVHD prophylaxis in r/r AML patients who received prior PD-1 antibodies before transplantation for the first time. Athough only four patients in the PTCy group could be evaluated, the results are encouraging. No grade 3-4 aGVHD, moderate-to-severe cGVHD, relapse, or death were observed with a median follow-up of 7.7 months. This indicates that the PTCy group seems to show an initiatory trend toward better outcomes when compared with the non-PTCy group. However, given the small sample size of this study, conducting a subgroup analysis would further reduce the number of cases per group, making meaningful statistical analysis challenging. Despite these limitations, the observed trends justify further investigation with an expanded sample size to confirm these initial findings.

To the best of our knowledge, previous studies among patients with AML and myelodysplastic syndromes (MDS) reported that the incidence of severe aGVHD (22%-26%) was higher than without the use of PTCy (13, 19). Similarly, our results in the patients without PTCy group (n=11) concurred with those, in which the incidence of grade 3-4 aGVHD is 18.2% (**Supplementary Table 2**). Previous retrospective investigations among patients with myeloid malignancies exposed to PD-1 inhibitor pretransplant mainly focused on HLA-matched transplants (13, 19). In our results, most of the patients (12/15, 80%) received peripheral blood grafts from HID, while the remaining 2 received from MSD, and 1 from a URD.

Notably, all 4 patients in the PTCy group survived in complete CR without severe toxicity and GVHD, which is rare after allo-HSCT for r/r AML. The addition of PTCy failed to increase transplantation-related mortality or the relapse rate after transplantation besides affecting hematopoietic reconstitution. Our study preliminarily corroborated the findings of Oran et al., indicating a consensus on the use of PTCy as GVHD prophylaxis improves outcomes of patients exposed to anti-PD-1 pretransplant (19, 24, 25). Previous preclinical models also suggested that inhibition of the PD-1 axis around the time of allo-HSCT augments the activity of alloreactive T cells and impairs the expansion of T regulatory cells, resulting in both increased immune-related toxicity and anti-tumor activity (17, 45–47). PTCy may be able to restore Treg and effector T cell homeostasis, thereby lowering the risk of acute GVHD (17). All the aforementioned findings support the results observed in this study. Though our results align with the aspects mentioned by Oran et al., discrepancies arise in terms of different disease diagnoses and risk stages, allograft donor source, GVHD prophylaxis regimen containing both PTCy and ATG and the span of follow-up period.

Our study innovatively contributes by the combination of PTCy and ATG for GVHD prophylaxis in r/r AML patients receiving PD1 antibodies pre-transplantation, which distinguishes it from the previous findings of Wang et al. who focused on the combination of PTCy and ATG as an effective strategy for GVHD prevention in haploidentical patients (48). However, six patients with classic HL exposed to PD-1 blockade before allo-HSCT who received both PTCy and ATG had exceptionally poor outcomes with a very high risk of NRM (23). One of the potential reasons may be associated with different primary disease types and their inherent characteristics. Therefore, further mechanism explorations and prospective clinical trials are needed to validate and refine these preliminary findings. Moreover, the shorter number of chemotherapy cycles (median number of prior systemic therapy cycle: 3; median number of tislelizumab + HMA + CAG treatment cycle: 1) may be associated with less endothelial dysfunction and tissue injury, thereby contributing to lower GVHD. In addition, there is evidence that a longer interval from anti-PD-1 to allo-HSCT may be associated with less frequent severe aGVHD and the International consensus guidelines have recommended a 6-week washout period between PD-1 blockade therapy and transplant (23, 49). Consequently, the relatively longer interval from the last dose of anti-PD-1 antibody to the transplantation (median interval > 10 weeks) in the current study may also explain the low incidence of severe aGVHD.

Our study had a few limitations that should be taken into consideration before arriving at a conclusion. Firstly, due to the sample size limitation and short follow-up, it was feasible to perform statistical test analysis which might affect the conclusions drawn. Secondly, there is a lack of studies investigating the possible mechanisms by which PTCy mitigates the risk of aGVHD in patients exposed to anti-PD-1 prior to HSCT, making correlations difficult. Hence, these findings can be considered preliminary and there is an unmet need to verify the results with a larger sample size to accurately identify the benefits of allo-HSCT after tislelizumab + HMA + CAG therapy and the impact of the immunological profile of patients post-transplant. Perhaps most importantly, given our finding that the combination of reduced-dose PTCy and ATG-based GVHD prophylaxis is associated with significant improvements in NRM and relapse, we highly recommend this strategy to be further studied for patients with r/r AML receiving allo-HSCT after PD-1 blockade. A prospective single-arm clinical study evaluating this regimen has already been registered and is ongoing (ClinicalTrials.gov identifier NCT06238245).

## Data Availability

The raw data supporting the conclusions of this article will be made available by the authors, without undue reservation.

## References

[1] DeWolf S Tallman MS. How I treat relapsed or refractory AML. Blood. (2020) 136:1023–32. doi: 10.1182/blood.2019001982 PMC7453152 32518943

[2] Iwai Y Ishida M Tanaka Y Okazaki T Honjo T Minato N. Involvement of PD-L1 on tumor cells in the escape from host immune system and tumor immunotherapy by PD-L1 blockade. Proc Natl Acad Sci U S A. (2002) 99:12293–7. doi: 10.1073/pnas.192461099 PMC129438 12218188

[3] Zou W Wolchok JD Chen L. PD-L1 (B7-H1) and PD-1 pathway blockade for cancer therapy: Mechanisms, response biomarkers, and combinations. Sci Transl Med. (2016) 8:328rv4. doi: 10.1126/scitranslmed.aad7118 PMC4859220 26936508

[4] Xu X Zhang W Xuan L Yu Y Zheng W Tao F. PD-1 signalling defines and protects leukaemic stem cells from T cell receptor-induced cell death in T cell acute lymphoblastic leukaemia. Nat Cell Biol. (2023) 25:170–82. doi: 10.1038/s41556-022-01050-3 36624186

[5] Atanackovic D Luetkens T. Biomarkers for checkpoint inhibition in hematologic Malignancies. Semin Cancer Biol. (2018) . 52:198–206. doi: 10.1016/j.semcancer.2018.05.005 29775689

[6] Li Z Philip M Ferrell PB. Alterations of T-cell-mediated immunity in acute myeloid leukemia. Oncogene. (2020) 39:3611–9. doi: 10.1038/s41388-020-1239-y PMC7234277 32127646

[7] Zhou Q Munger ME Highfill SL Tolar J Weigel BJ Riddle M. Program death-1 signaling and regulatory T cells collaborate to resist the function of adoptively transferred cytotoxic T lymphocytes in advanced acute myeloid leukemia. Blood. (2010) 116:2484–93. doi: 10.1182/blood-2010-03-275446 PMC2953885 20570856

[8] Williams P Basu S Garcia-Manero G Hourigan CS Oetjen KA Cortes JE. The distribution of T-cell subsets and the expression of immune checkpoint receptors and ligands in patients with newly diagnosed and relapsed acute myeloid leukemia. Cancer. (2019) 125:1470–81. doi: 10.1002/cncr.31896 PMC6467779 30500073

[9] Chen C Liang C Wang S Chio CL Zhang Y Zeng C. Expression patterns of immune checkpoints in acute myeloid leukemia. J Hematol Oncol. (2020) 13:28. doi: 10.1186/s13045-020-00853-x 32245463 PMC7118887

[10] Zeidan AM Boss I Beach CL Copeland WB Thompson E Fox BA. A randomized phase 2 trial of azacitidine with or without durvalumab as first-line therapy for older patients with AML. Blood Adv. (2022) 6:2219–29. doi: 10.1182/bloodadvances.2021006138 PMC9006260 34933333

[11] Saxena K Herbrich SM Pemmaraju N Kadia TM DiNardo CD Borthakur G. A phase 1b/2 study of azacitidine with PD-L1 antibody avelumab in relapsed/refractory acute myeloid leukemia. Cancer. (2021) 127:3761–71. doi: 10.1002/cncr.33690 34171128

[12] Daver N Garcia-Manero G Basu S Boddu PC Alfayez M Cortes JE. Efficacy, safety, and biomarkers of response to azacitidine and nivolumab in relapsed/refractory acute myeloid leukemia: A nonrandomized, open-label, phase II study. Cancer Discovery. (2019) 9:370–83. doi: 10.1158/2159-8290.CD-18-0774 PMC6397669 30409776

[13] Ravandi F Assi R Daver N Benton CB Kadia T Thompson PA. Idarubicin, cytarabine, and nivolumab in patients with newly diagnosed acute myeloid leukaemia or high-risk myelodysplastic syndrome: a single-arm, phase 2 study. Lancet Haematol. (2019) 6:e480–8. doi: 10.1016/S2352-3026(19)30114-0 PMC6778960 31400961

[14] Gao XN Su YF Li MY Jing Y Wang J Xu L. Single-center phase 2 study of PD-1 inhibitor combined with DNA hypomethylation agent + CAG regimen in patients with relapsed/refractory acute myeloid leukemia. Cancer Immunol Immunother. (2023) 72:2769–82. doi: 10.1007/s00262-023-03454-y PMC10991359 37166484

[15] Hoos A Eggermont AM Janetzki S Hodi FS Ibrahim R Anderson A. Improved endpoints for cancer immunotherapy trials. J Natl Cancer Inst. (2010) 102:1388–97. doi: 10.1093/jnci/djq310 PMC2943524 20826737

[16] Merryman RW Armand P. Immune checkpoint blockade and hematopoietic stem cell transplant. Curr Hematol Malig Rep. (2017) 12:44–50. doi: 10.1007/s11899-017-0362-5 28155012

[17] Ikegawa S Meguri Y Kondo T Sugiura H Sando Y Nakamura M. PTCy ameliorates GVHD by restoring regulatory and effector T-cell homeostasis in recipients with PD-1 blockade. Blood Adv. (2019) 3:4081–94. doi: 10.1182/bloodadvances.2019000134 PMC6963247 31821459

[18] Merryman RW Kim HT Zinzani PL Carlo-Stella C Ansell SM Perales MA. Safety and efficacy of allogeneic hematopoietic stem cell transplant after PD-1 blockade in relapsed/refractory lymphoma. Blood. (2017) 129:1380–8. doi: 10.1182/blood-2016-09-738385 PMC5345733 28073785

[19] Oran B Garcia-Manero G Saliba RM Alfayez M Al-Atrash G Ciurea SO. Posttransplantation cyclophosphamide improves transplantation outcomes in patients with AML/MDS who are treated with checkpoint inhibitors. Cancer. (2020) 126:2193–205. doi: 10.1002/cncr.32796 32125707

[20] Ijaz A Khan AY Malik SU Faridi W Fraz MA Usman M. Significant Risk of Graft-versus-Host Disease with Exposure to Checkpoint Inhibitors before and after Allogeneic Transplantation. Biol Blood Marrow Transplant. (2019) 25:94–9. doi: 10.1016/j.bbmt.2018.08.028 PMC6310648 30195074

[21] Armand P Engert A Younes A Fanale M Santoro A Zinzani PL. Nivolumab for relapsed/refractory classic hodgkin lymphoma after failure of autologous hematopoietic cell transplantation: extended follow-up of the multicohort single-arm phase II checkMate 205 trial. J Clin Oncol. (2018) 36:1428–39. doi: 10.1200/JCO.2017.76.0793 PMC6075855 29584546

[22] Saberian C Abdel-Wahab N Abudayyeh A Rafei H Joseph J Rondon G. Post-transplantation cyclophosphamide reduces the incidence of acute graft-versus-host disease in patients with acute myeloid leukemia/myelodysplastic syndromes who receive immune checkpoint inhibitors after allogeneic hematopoietic stem cell transplantation. J Immunother Cancer. (2021) 9:e001818. doi: 10.1136/jitc-2020-001818 33637601 PMC7919586

[23] Merryman RW Castagna L Giordano L Ho VT Corradini P Guidetti A. Allogeneic transplantation after PD-1 blockade for classic Hodgkin lymphoma. Leukemia. (2021) 35:2672–83. doi: 10.1038/s41375-021-01193-6 33658659

[24] De Philippis C Legrand-Izadifar F Bramanti S Giordano L Montes de Oca C Duléry R. Checkpoint inhibition before haploidentical transplantation with posttransplant cyclophosphamide in Hodgkin lymphoma. Blood Adv. (2020) 4:1242–9. doi: 10.1182/bloodadvances.2019001336 PMC7160255 32227210

[25] Paul S Zahurak M Luznik L Ambinder RF Fuchs EJ Bolaños-Meade J. Non-myeloablative allogeneic transplantation with post-transplant cyclophosphamide after immune checkpoint inhibition for classic hodgkin lymphoma: A retrospective cohort study. Biol Blood Marrow Transplant. (2020) 26:1679–88. doi: 10.1016/j.bbmt.2020.06.012 PMC7486273 32592857

[26] Döhner H Wei AH Appelbaum FR Craddock C DiNardo CD Dombret H. Diagnosis and management of AML in adults: 2022 recommendations from an international expert panel on behalf of the ELN. Blood. (2022) 140:1345–77. doi: 10.1182/blood.2022016867 35797463

[27] DHner H Estey E Grimwade D Amadori S Appelbaum FR Büchner T. Diagnosis and management of AML in adults: 2017 ELN recommendations from an international expert panel. BLOOD. (2017) 129:424–47. doi: 10.1182/blood-2016-08-733196 PMC5291965 27895058

[28] Arber DA Orazi A Hasserjian RP Borowitz MJ Calvo KR Kvasnicka HM. International Consensus Classification of Myeloid Neoplasms and Acute Leukemias: integrating morphologic, clinical, and genomic data. Blood. (2022) 140:1200–28. doi: 10.1182/blood.2022015850 PMC9479031 35767897

[29] Sorror ML Maris MB Storb R Baron F Sandmaier BM Maloney DG. Hematopoietic cell transplantation (HCT)-specific comorbidity index: a new tool for risk assessment before allogeneic HCT. Blood. (2005) 106:2912–9. doi: 10.1182/blood-2005-05-2004 PMC1895304 15994282

[30] Armand P Kim HT Logan BR Wang Z Alyea EP Kalaycio ME. Validation and refinement of the Disease Risk Index for allogeneic stem cell transplantation. Blood. (2014) 123:3664–71. doi: 10.1182/blood-2014-01-552984 PMC4047501 24744269

[31] Vigorito AC Campregher PV Storer BE Carpenter PA Moravec CK Kiem HP. Evaluation of NIH consensus criteria for classification of late acute and chronic GVHD. Blood. (2009) 114:702–8. doi: 10.1182/blood-2009-03-208983 PMC2713471 19470693

[32] Holtan SG DeFor TE Lazaryan A Bejanyan N Arora M Brunstein CG. Composite end point of graft-versus-host disease-free, relapse-free survival after allogeneic hematopoietic cell transplantation. Blood. (2015) 125:1333–8. doi: 10.1182/blood-2014-10-609032 PMC4335084 25593335

[33] Wang H Wang N Wang L Du J Li F Shao Y. Targeted dosing of anti-thymocyte globulin in adult unmanipulated haploidentical peripheral blood stem cell transplantation: A single-arm, phase 2 trial. Am J Hematol. (2023) 98:1732–41. doi: 10.1002/ajh.27068 37706580

[34] Lu DP Dong L Wu T Huang XJ Zhang MJ Han W. Conditioning including antithymocyte globulin followed by unmanipulated HLA-mismatched/haploidentical blood and marrow transplantation can achieve comparable outcomes with HLA-identical sibling transplantation. Blood. (2006) 107:3065–73. doi: 10.1182/blood-2005-05-2146 16380454

[35] [The guidelines for diagnosis and treatment of relapse /refractory acute myelogenous leukemia in China (2023)]. Zhonghua Xue Ye Xue Za Zhi. (2023) 44:713–6. doi: 10.3760/cma.j.issn.0253-2727.2023.09.002 PMC10630579 38049313

[36] DiNardo CD Lachowiez CA Takahashi K Loghavi S Xiao L Kadia T. Venetoclax combined with FLAG-IDA induction and consolidation in newly diagnosed and relapsed or refractory acute myeloid leukemia. J Clin Oncol. (2021) 39:2768–78. doi: 10.1200/JCO.20.03736 PMC8407653 34043428

[37] Jin H Zhang Y Yu S Du X Xu N Shao R. Venetoclax combined with azacitidine and homoharringtonine in relapsed/refractory AML: A multicenter, phase 2 trial. J Hematol Oncol. (2023) 16:42. doi: 10.1186/s13045-023-01437-1 37120593 PMC10149010

[38] Wang H Mao L Yang M Qian P Lu H Tong H. Venetoclax plus 3 + 7 daunorubicin and cytarabine chemotherapy as first-line treatment for adults with acute myeloid leukaemia: a multicentre, single-arm, phase 2 trial. Lancet Haematol. (2022) 9:e415–24. doi: 10.1016/S2352-3026(22)00106-5 35512726

[39] Liu Y Zhu L Lv Z Mao L Hu C Wang J. Venetoclax plus azacitidine and LDAC induced high response rates in acute myeloid leukaemia in routine clinical practice. Br J Haematol. (2023) 201:995–9. doi: 10.1111/bjh.18788 36999439

[40] Kadia TM Reville PK Borthakur G Yilmaz M Kornblau S Alvarado Y. Venetoclax plus intensive chemotherapy with cladribine, idarubicin, and cytarabine in patients with newly diagnosed acute myeloid leukaemia or high-risk myelodysplastic syndrome: a cohort from a single-centre, single-arm, phase 2 trial. Lancet Haematol. (2021) 8:e552–61. doi: 10.1016/S2352-3026(21)00192-7 PMC8884174 34329576

[41] Cortes J Jonas BA Schiller G Mims A Roboz GJ Wei AH. 0Olutasidenib in post-venetoclax patients with mutant isocitrate dehydrogenase 1 (mIDH1) acute myeloid leukemia (AML). Leuk Lymphoma. (2024) 65:1145–52. doi: 10.1080/10428194.2024.2333451 38538632

[42] Short NJ Daver N Dinardo CD Kadia T Nasr LF Macaron W. Azacitidine, venetoclax, and gilteritinib in newly diagnosed and relapsed or refractory FLT3-mutated AML. J Clin Oncol. (2024) 42:1499–508. doi: 10.1200/JCO.23.01911 PMC11095865 38277619

[43] Daver N Perl AE Maly J Levis M Ritchie E Litzow M. Venetoclax plus gilteritinib for FLT3-mutated relapsed/refractory acute myeloid leukemia. J Clin Oncol. (2022) 40:4048–59. doi: 10.1200/JCO.22.00602 PMC9746764 35849791

[44] Lachowiez CA Loghavi S Zeng Z Tanaka T Kim YJ Uryu H. A phase ib/II study of ivosidenib with venetoclax ± Azacitidine in IDH1-mutated myeloid Malignancies. Blood Cancer Discovery. (2023) 4:276–93. doi: 10.1158/2643-3230.BCD-22-0205 PMC10320628 37102976

[45] Michonneau D Sagoo P Breart B Garcia Z Celli S Bousso P. The PD-1 axis enforces an anatomical segregation of CTL activity that creates tumor niches after allogeneic hematopoietic stem cell transplantation. Immunity. (2016) 44:143–54. doi: 10.1016/j.immuni.2015.12.008 26795248

[46] Saha A Aoyama K Taylor PA Koehn BH Veenstra RG Panoskaltsis-Mortari A. Host programmed death ligand 1 is dominant over programmed death ligand 2 expression in regulating graft-versus-host disease lethality. Blood. (2013) 122:3062–73. doi: 10.1182/blood-2013-05-500801 PMC3811178 24030385

[47] Blazar BR Carreno BM Panoskaltsis-Mortari A Carter L Iwai Y Yagita H. Blockade of programmed death-1 engagement accelerates graft-versus-host disease lethality by an IFN-gamma-dependent mechanism. J Immunol. (2003) 171:1272–7. doi: 10.4049/jimmunol.171.3.1272 12874215

[48] Wang Y Wu DP Liu QF Xu LP Liu KY Zhang XH. Low-dose post-transplant cyclophosphamide and anti-thymocyte globulin as an effective strategy for GVHD prevention in haploidentical patients. J Hematol Oncol. (2019) 12:88. doi: 10.1186/s13045-019-0781-y 31481121 PMC6724335

[49] Herbaux C Merryman R Devine S Armand P Houot R Morschhauser F. Recommendations for managing PD-1 blockade in the context of allogeneic HCT in Hodgkin lymphoma: taming a necessary evil. Blood. (2018) 132:9–16. doi: 10.1182/blood-2018-02-811174 29720488

