# Supplementary material for: Anti-PD-1 combined with hypomethylating agent and CAG regimen bridging to allogeneic hematopoietic stem cell transplantation: a novel strategy for relapsed/refractory acute myeloid leukemia
Source: Front Immunol. 2024 Aug 16;15:1409302. doi: 10.3389/fimmu.2024.1409302 (PMC11361969; doi:10.3389/fimmu.2024.1409302)
Supplement: Supplementary file 1 [file DataSheet1.pdf]

Supplement Table 1. Toxicity, outcomes, and deaths of 15 patients during transplantation.

Toxicity and outcomes

	Event/No.	Estimate (95% CI)
Acute GVHD (100-days)	7/15 (46.7%)	46.7% (27.2%-80.2%)
Grade 1-2	5/15 (33.3%)	33.3% (16.3-68.2%)
Grade 3-4	2/15 (13.3%)	13.3% (3.7%-48.5%)
Chronic GVHD (2-year)	4/12 (33.3%)	40.9% (18.9%-88.6%)
Moderate-to-severe cGVHD	1/12 (8.3%)	10% (1.6%-64.2%)
CMV Infection (2-year)	7/15 (46.7%)	47% (27%-80%)
CMV DNAemia	6/15 (40%)	40% (22%-74%)
CMV cystitis	1/15 (6.7%)	7% (1%-44%)
CMV Pneumonia	1/15 (6.7%)	8% (1%-51%)
EBV DNAemia (2-year)	5/15 (33.3%)	43% (16%-68%)
PTLD (2-year)	1/15 (6.7%)	13% (2%-79%)
TA-TMA (2-year)	1/15 (6.7%)	7% (1%-45%)
2-year OS	6/15 (40%)	54% (26.1%-81.9%)
2-year CIR	3/15 (20%)	22.5% (8.1%-62.2%)
2-year NRM	3/15 (20%)	22.3% (8.1%-61.7%)
2-year GRFS rate	7/15 (46.7%)	48.6% (20.9%-76.3%)

Deaths

No.	Age	Donor source	Interval between PD-1 and HSCT, month	Day of months	Cause of death	Complications
6	52	Haploidentical	1.7	1.2	Grade 4 gastrointestinal aGVHD	Acute kidney injury, drug-induced liver injury, grade 3-4 gastrointestinal aGVHD intracranial hemorrhage, gastrointestinal hemorrhage
8	30	Haploidentical	2.4	14.8	LOSP	Grade 3 gastrointestinal aGVHD, grade 1 hepatic aGVHD, drug-induced liver injury, hemorrhagic cystitis, LOSP
12	56	Haploidentical	2.3	2.1	Pulmonary infection	Grade 1 skin aGVHD, peritoneal effusion, MODS, TA-TMA

GVHD, graft-versus-host disease; CMV, Cytomegalovirus; EBV, Epstein-Barr virus; PTLD: Post-transplant lymphoproliferative disorder; TA-TMA, transplant-associated thrombotic microangiopathy; OS, overall survival; CIR, cumulative incidence rate; NRM, non-relapse mortality; GRFS, GVHD-free/relapse-free survival; LOSP, late-onset severe pneumonia; MODS, multiple organ dysfunction syndrome.

Supplement Table 2. Transplantation outcomes of transplantation by GVHD prophylaxis.

	All Patients N = 15		No PTCy Group N = 11		PTCy Group N = 4	
	Event/No.	Estimate (95% CI)	Event/No.	Estimate (95% CI)	Event/No.	Estimate (95% CI)
Acute GVHD (100-days)	7/15	46.7% (27.2%-80.2%)	4/11	36.3% (16.6%-79.5%)	3/4	75% (42.6%-100%)
Grade II-IV	6/15	40% (21.5-74.3%)	3/11	27.3% (10.4%-71.6%)	3/4	75% (42.6%-101%)
Grade III-IV	2/15	13.3% (3.7%-48.5%)	2/11	18.2% (5.2%-63.7%)	0/4	0%
Chronic GVHD (2-year)	4/12	40.9% (18.9%-88.6%)	3/8	41.7% (17.4%-99.7%)	1/4	50% (12.5%-100%)
Moderate-to-severe cGVHD	1/12	10% (1.6%-64.2%)	1/8	14.3% (2.3%-8.8%)	0/4	0%
2-year OS	6/15	54% (26.1%-81.9%)	6/11	40% (9.6%-70.4%)	0/4	100%
2-year CIR	3/15	22.5% (8.1%-62.2%)	3/11	28.8% (11%-75.4%)	0/4	0%
2-year NRM	3/15	22.3% (8.1%-61.7%)	3/11	28.6% (10.9%-74.9%)	0/4	0%
1-year GRFS rate	7/15	48.6% (20.9%-76.3%)	7/11	67.3% (43.5%-100%)	0/4	100%

GVHD, graft-versus-host disease; OS, overall survival; CIR, cumulative incidence rate; NRM, non-relapse mortality; GRFS, GVHD-free/relapse-free survival.

Supplement Figure 1.

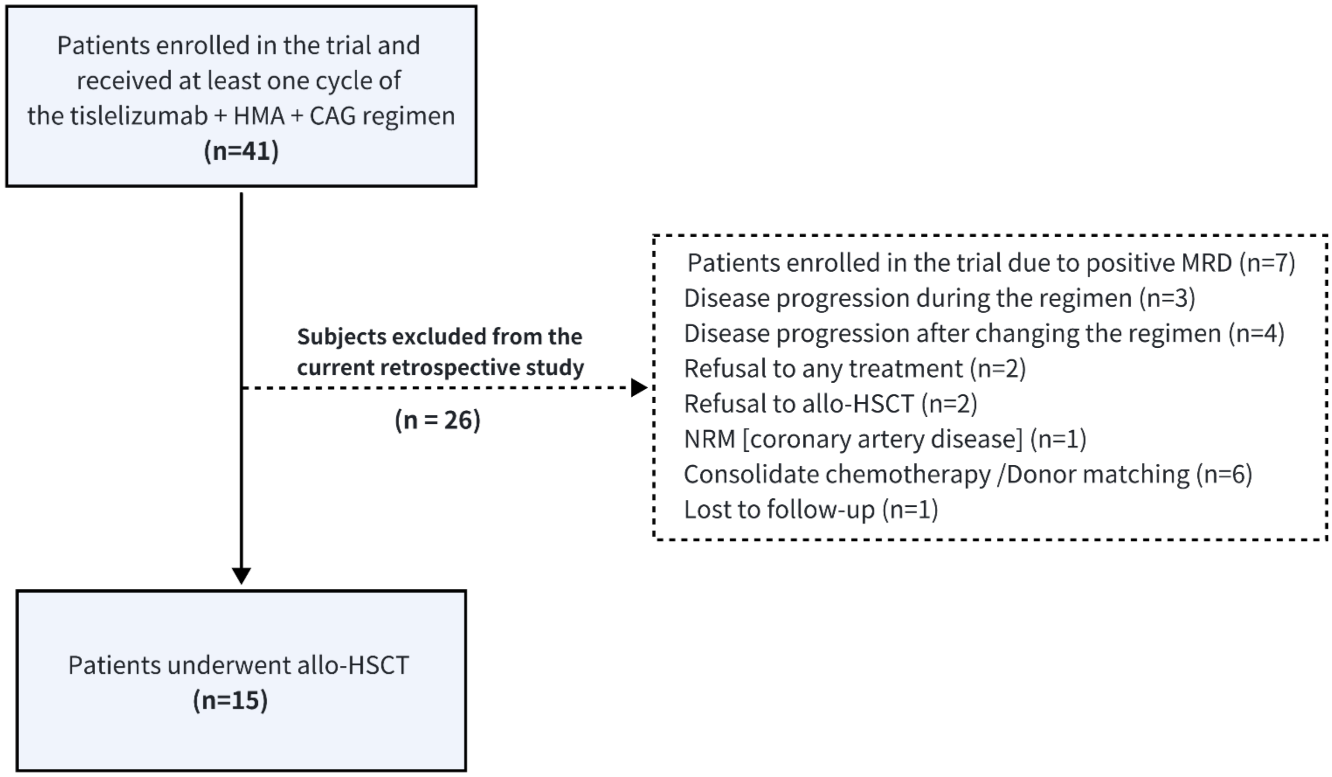

Figure 1. The schematic diagram of the entire clinical study design plan.

HMA: hypomethylating agent; CAG: cytarabine, aclarubicin/ idarubicin, granulocyte colony-stimulating factor; MRD, measurable residual disease; NRM, non-relapse mortality; allo-HSCT: allogeneic hematopoietic stem cell transplant.
